# Health-related quality of life of Kuwaiti adults living with diabetes

**DOI:** 10.3389/fpubh.2023.1085928

**Published:** 2023-03-23

**Authors:** Maryam S. Alowayesh, Syed Mohamed Aljunid, Afaf Aladsani, Thamer Alessa, Abdulnabi Alattar, Dherar Alroudhan

**Affiliations:** ^1^Department of Pharmacy Practice, Faculty of Pharmacy, Kuwait University, Kuwait City, Kuwait; ^2^Department of Health Policy and Management, Faculty of Public Health, Kuwait University, Kuwait City, Kuwait; ^3^International Medical University, Kuala Lumpur, Malaysia; ^4^Al-Sabah Hospital, Ministry of Health, Kuwait City, Kuwait; ^5^Jaber Hospital, Ministry of Health, Kuwait City, Kuwait; ^6^Al-Amiri Hospital, Ministry of Health, Kuwait City, Kuwait; ^7^Mubarak Hospital, Ministry of Health, Salmiya, Kuwait

**Keywords:** quality of life, EQ-5D, Kuwait, diabetes, HRQoL

## Abstract

Diabetes is known to compromise patients' health-related quality of life (HRQoL). It is important to understand the HRQoL of Kuwaiti nationals living with diabetes and identify factors that negatively affect it. This study included 1,182 Kuwaiti nationals with diabetes, aged 18–80 years. Patients' demographic and HRQoL information was collected using the EuroQol EQ-5D-5L instrument. Mean values for each EQ-5D subscale were worst for mobility (1.9) and pain/discomfort (1.8). Most patients reported having no problems in self-care (84.4%). Older adults were most likely to report severe problems with mobility (*p* < 0.001). Females were more likely to report severe problems with anxiety and depression than males (*p* < 0.006). The EQ visual analog scale (EQ VAS), which reports perceived overall health on a 0–100 scale, showed a mean of 74.7 (±19.6). Patients with comorbidities and/or complications perceived their health to be worse, with VAS scores significantly lower (*p* < 0.001). Other factors that negatively affected VAS scores were being female, obesity, insulin usage, and lower levels of education. In conclusion, patients with diabetes who have comorbidities and/or complications perceived their health to be worse. Further research is required to evaluate if current diabetes management interventions help improve patients' HRQoL.

## 1. Introduction

Globally, the prevalence of diabetes and its complications is growing, which places a considerable burden on national healthcare systems' resources and expenditures. The International Diabetes Federation (IDF) estimated the 2019 worldwide prevalence of diabetes in adults (aged 20–79 years) to be 9.3% (463 million people) (1). The IDF's estimated prevalence of diabetes in 21 countries and territories of the Middle East and North Africa (MENA) region, which includes Kuwait, is higher, at 12.2% (1). A study published in 2020 but using data from 2014 estimated the prevalence of diabetes in Kuwait to be 21.8%. The same study estimated the prevalence of pre-diabetes (impaired fasting glucose or impaired glucose tolerance) at 11.1% among Kuwait citizens (2).

Patients living with diabetes often have severe and long-term complications or comorbidities, which add layers of complexity to patients' health management involving additional medications, potential side effects, and costs (1).

Increasingly, healthcare systems are recognizing that diabetes is a complicated disease, with health-related quality of life (HRQoL) factors contributing to the clinical aspects of the successful management of diabetes treatment. HRQoL is defined in many ways, but in general, it involves a wide range of health- or treatment-related factors from three broad domains: physical, psychological, and social functioning (3). In the 2020 Standards of Medical Care in Diabetes, the American Diabetes Association (ADA) states: “Effective behavior management and psychological wellbeing are foundational to achieving treatment goals for people with diabetes. Hence understanding how patients' health related quality of life is effected by diabetes is crucial. Essential to achieving these goals are diabetes self-management education and support (DSMES), medical nutrition therapy (MNT), routine physical activity, smoking cessation counseling when needed, and psychosocial care (4). This holistic approach in patient management improves patient's quality of life (4).”

Understanding how diabetes and its comorbidities and complications affect the HRQoL of people living with diabetes is essential. It has been reported that patients suffering from diabetes complications are more prone to diabetes distress and depression, which will affect their health related quality of life negatively (4). However, diabetes-related HRQoL data for the Kuwaiti population is limited. Our study examined the impact of diabetes and its related comorbidities and complications on patient HRQoL in Kuwaiti nationals living with diabetes. This study may help in advancing Kuwait's healthcare goals to improve treatment, standards of care, outcomes, and costs of treating patients with diabetes.

## 2. Materials and methods

### 2.1. Study design

For this cross-sectional study, patients from all six Kuwait governorates, including one hospital and two primary care centers from each governorate, were recruited. Centers were selected if they were in areas with high populations of Kuwaiti nationals and if the center's administration were willing to participate in the study. In addition, two primary care centers with diabetes clinics were selected from a list of clinics provided by the Ministry of Health (MOH). The clinics were selected according to these criteria. Clinics that have a diabetes clinic, high number of Kuwaiti patients, and willing to collaberate. Due to a shortage of resources, we approached all eligible patients that entered the clinics on data collection days, meaning convenient sampling rather than systematic random sampling was used.

### 2.2. Sampling frame

For the sample to be representative of the Kuwaiti population, we used a multi-stage stratified sampling method. The first stage was the governorates. The second stage was the health facilities (i.e., hospital and primary care centers). The weight of the strata are accounted for in the sampling; in this case, the strata were the governorates. It was estimated that 741,648 Kuwaiti adults were living in all six governorates, according to the June 2018 census from the public authority of civil information. To determine the weights, we divided the number of Kuwaiti adults in each governorate by the lowest number of Kuwaiti adults found in the smallest governorate (Mubarak Alkabeer). To obtain the number for each governorate, the resulting weights were multiplied by our target total sample size (1,200), as shown in Table 1. Due to a lack of a priori information on the distribution of diabetic patients between primary and secondary care, the number for each governorate was divided equally between the hospital (50%) and the two primary care centers (50%).

**Table 1:** Numbers of patients recruited from each governorate.

**Governorate**	**Kuwait adult population**	**Study sample**	**Primary care**	**Secondary care**
Capital	161,203	260	130	130
Hawali	140,894	228	114	114
Ahmadi	147,017	238	119	119
Farwaniyah	102,610	166	133	133
Aljahra	96,568	156	128	128
Mubarak Al-Kabeer	93,446	152	126	126
Total	741,648	1,200	600	600

### 2.3. Patient selection

#### 2.3.1. Eligibility criteria

Kuwaiti adults (aged 18–80) diagnosed with either diabetes type 1 or type 2, with or without complications, were eligible and recruited for this study. The rationale of the age cut off was that we did not want to have pediatric population, because diabetes representation in pediatrics is different, hence above 18. The 80 upper limit was because patients above 80 years old are more likely to have memory issues and clinically complex cases due to age-related comorbidities not due to diabetes.

#### 2.3.2. Inclusion criteria

Only Kuwaiti nationals diagnosed with diabetes during or before January 2018 were included.

#### 2.3.3. Exclusion criteria

Non-Kuwaitis were excluded because they do not have the same access to care as Kuwaitis. Patients with dementia or who were not mentally capable of completing the interview were excluded. Because gestational diabetes is primarily a temporary type of diabetes, females with gestational diabetes were excluded.

### 2.4. Data collection form

For this study, a data collection form was created through consultation with four diabetologists, a health economist, and five consultants specializing in diabetes complications (a cardiologist, a nephrologist, a neurologist, an ophthalmologist, and a vascular surgeon). The form was used to collect each patient's demographic data and clinical data such as diabetes type, onset and duration of disease, most recent HbA1c level, comorbidities, and complications. The EQ-5D-5L tool was attached to the form.

### 2.5. Data collection process

Data collection period started in August 2019 and ended by end of December 2019. The data collector approached patients waiting for their appointments in the seating area of diabetes outpatient clinics in hospitals and primary care centers. To decrease the convenient sampling bias, all patients in the seating area were approached, regardless of friendliness or willingness to participate. The data collector first screened each patient for eligibility by asking if the patient had been diagnosed with diabetes for at least 1 year, were an adult aged 18–80 years, and were a Kuwaiti national. If the patient was eligible and willing to participate, the data collector obtained consent by asking the patient to sign a consent form, then collected the patient's demographic and HRQoL data. Next, the data collector accompanied the patient to the entrance of the clinic and informed the physician that the patient had consented to participate in the study. The data collector then gave the clinical data collection form to the physician to complete for that patient. The physician collected the clinical data either from the patient chart or, if the information was not available in the chart, by asking the patient directly. After completing the form, the physician handed it to the data collector.

### 2.6. Definitions

Comorbidities were defined as patient diseases not caused by diabetes (e.g., hypertension and Dyslipidemia). Dyslipidemia is defined as elevated lipid levels in the blood. Complications were defined as patient diseases caused by diabetes [e.g., diabetic nephropathy, retinopathy, neuropathy, peripheral vascular diseases, and cardiovascular (i.e. coronary diseases) and cerebrovascular diseases (i.e. stroke)]. Physicians confirmed the presence of complications and comorbidities by reviewing the patient's medical record and by asking the patient questions, such as whether they visit a specialized doctor to monitor their complications, if they take medications for complications, or if they are experiencing any symptoms of complications.

### 2.7. Quality of life data

Data on patient HRQoL was collected using the EuroQol 5-dimension 5-level (EQ-5D-5L) standardized instrument for measuring health-related quality of life. The EQ-5D-5L instrument consists of two parts. The first part is the EQ-5D-5L descriptive system, which uses sub-scales to measure five aspects of quality of life: mobility, self-care, usual activities, pain/discomfort, and anxiety/depression. Respondents provide a score from 1 to 5 for each sub-scale, where 1 indicates no problems and 5 indicates extreme problems. The second part of the EQ-5D-5L instrument is the visual analog scale (EQ VAS). In this section, every patient was asked to rate their perceived health on a 100-point scale, with 0 being the worst health one can imagine and 100 being the best (5). The EQ-5D-5L plus VAS instrument was selected because it is widely used, user-friendly, and fast to complete. In addition, results are conducive to being converted to quality-adjusted life years (QALYs), making them readily available for comparisons with data from similar studies. In addition, the EQ-5D-5L and EQ VAS have a validated Arabic version, appropriate for use with the Kuwaiti population.

### 2.8. Ethical considerations

This study was given ethical approval by Kuwait University Health Sciences Center (KU-HSC) and Ministry of Health (MOH) Ethical Committee. Written informed consent was obtained from the patients who participated in the study.

### 2.9. Statistical analysis

We used mean and standard deviation to describe continuous variables, and we used counts and percentages to describe categorical variables. To examine the correlation between the VAS score and the EQ-5D-5L sub-scales, we used the Spearman correlation coefficient. To assess the impact of comorbidity and complication status on the HRQoL evaluation, a set of regression models adjusting for significant covariates was performed. Significance level was set at *p* < 0.05. IBM SPSS Statistics version 26 software was used to perform the statistical analysis.

The assumptions of both the linear regression and the ordinal logistic regression were checked to prove the data fit. For linear regression, a relationship was identified between each of the predictors used in the final model and the VAS score. The distribution of residuals for linear regression was approximately normal, and visual inspection of the plot of standardized residuals vs. standardized predicted values showed homoscedasticity. Testing the predictors proved no multicollinearity. Therefore, this method was determined to be applicable.

A major assumption for ordinal logistic regression is the assumption of proportional odds, which means each independent variable has an identical effect at each cumulative split of the ordinal dependent variable. To prove this assumption, a parallel test was performed. The initial test showed the assumption was violated for 4 of 5 sub-scales. However, considering that the share of patients providing the highest score on each of the sub-scales (5 of 5) was relatively low (no more than 2%), the decision was made to merge them with the patients who provided a score of 4. This made the last group more representative and avoided the issue of accommodating many empty cells in regression models due to few patients in that category. The merging of the two sub-scale groups resulted in improvement in parallel test results, with all models fitting the assumption of proportional odds.

To create multivariate models, predictors with a significant impact on the dependent variable were selected. The final model for VAS scores (**Table 5**) included gender, comorbidity and complication status, education level, and whether the patient was taking insulin. Other predictors including age, duration of disease, obesity, HbA1c score, and diabetes type provided insignificant impact on the VAS score after adjusting for all the covariates and therefore were not included in the final model.

## 3. Results

### 3.1. Demographic and clinical characteristics

The study included 1,182 patients, aged 18–80 years with a mean age of 56.3 (±13.1) years (Table 2). Most patients were females (65.3%) and were diagnosed with type 2 diabetes (92.0%). The duration of the disease was 13.3 (±8.7) years. One third of the patients (35.9%) had both comorbidities and complications, while 43.4% had only comorbidities and 4.8% had only complications. Only 15.9% patients did not have any comorbidities or complications. The most common comorbidities were hypertension (57.7%) and hyperlipidemia (64.0%). The most frequent complications were neuropathy (19.7%) and retinopathy (16.9%).

**Table 2:** Patients' socio-demographic and clinical characteristics.

**Parameter**	**Statistics**	***N* (%)**
**Socio-demographics**		
Gender	Male	404 (34.4%)
	Female	772 (65.6%)
Age (years)	Mean (SD)	56.3 (13.1)
Education	Illiterate	150 (13.2%)
	Elementary/intermediate/secondary	501 (44.1%)
	Diploma/university	486 (42.7%)
Marital status	Single	111 (9.8%)
	Married	839 (73.9%)
	Divorced/widowed	185 (16.3%)
Occupation	Employed	360 (30.4%)
	Student	26 (2.2%)
	Housewife	359 (30.4%)
	Retired/unemployed	397 (33.6%)
	N/A	40 (3.4%)
Decreased workload due to medical reasons	Yes	18 (1.5%)
	No	221 (18.7%)
	N/A	943 (79.8%)
**Disease characteristics**		
Duration of disease (years)	Mean (SD)	13.3 (8.7)
Diabetes type	Type 1	92 (8.0%)
	Type 2	1,058 (92%)
Comorbidity and complication	No complications or comorbidities	188 (15.9%)
	Only comorbidities	513 (43.4%)
	Only complications	57 (4.8%)
	Both complications and comorbidities	424 (35.9%)
Comorbidities (detailed)	None	245 (20.7%)
	Hypertension	682 (57.7%)
	Hyperlipidemia	757 (64.0%)
	Other	208 (17.6%)
Complications (detailed)	No complications	701 (59.3%)
	Peripheral neuropathy	233 (19.7%)
	Retinopathy	200 (16.9%)
	Nephropathy	165 (14.0%)
	Cardiovascular disease	116 (9.8%)
	Peripheral vascular disease	11 (0.9%)
Most recent HbA1c (%)	Mean (SD)	8.1 (5.3)
	<7, controlled	296 (25.0%)
	7–8, nearly controlled	287 (24.3%)
	>8, not controlled	465 (39.3%)
	N/A	134 (11.3%)
Insulin therapy	Yes	540 (45.7%)
	No	642 (54.3%)
**Overall health status and behavior**		
Patient weight (kg)	Mean (SD)	84.8 (17.7)
Patient height (cm)	Mean (SD)	161.6 (10.4)
BMI (kg/m^2^)	Mean (SD)	32.7 (7.5)
Obesity	Non obese (BMI <30)	350 (36.7%)
	Obese (BMI ≥30)	603 (63.3%)
BMI categories	Underweight (BMI <18 kg/m^2^)	6 (0.6%)
	Normal weight (BMI 18.5–24.9 kg/m^2^)	91 (9.5%)
	Overweight (BMI 25–29.9 kg/m^2^)	253 (26.5%)
	Obese (BMI >30 kg/m^2^)	603 (63.3%)
Smoking status	Current	153 (12.9%)
	Former	102 (8.6%)
	Never	863 (73.0%)
	N/A	64 (5.4%)

BMI, body mass index; HbA1c, hemoglobin A1c; SD, standard deviation; N/A, not answered.

Almost two thirds of the patients (63.3%) were obese, with a mean body mass index (BMI) for all patients of 32.7 (±7.5). Fewer than half of the patients (45.7%) reported taking insulin. Only 25.0% of the study population had HbA1c levels <7.0% (controlled), almost the same number of patients (24.3%) had HbA1c levels of 7.0%−8.0% (nearly controlled), and over a third (39.3%) of patients had HbA1c levels >8.0% (not controlled).

The patients were almost equally distributed between those with secondary and those with higher education (44.1 and 42.7% respectively). About two thirds of the patients were not employed, with 31.8% being retired or resigned and another 34.4% not working.

### 3.2. Patients' reported health-related quality of life

Table 3 presents the mean scores of the EQ-5D-5L sub-scales. The mean values for separate EQ-5D sub-scales show the highest scores (worst) were for mobility (1.9 ± 1.1) and pain/discomfort (1.8 ± 1.1), which indicates these two aspects of HRQoL are the most problematic for patients. Anxiety/depression was the third-highest aspect reported (1.6 ± 1.0). Usual activities and self-care were assessed at lower scores (1.4 ± 0.9 and 1.3 ± 0.7, respectively), indicating they were considered less problematic by patients.. The overall VAS score for our study population, which reports perceived overall health on a 0–100 scale, showed a mean of 74.7 (±19.6). A comparison by comorbidity and complication status showed that the EQ-5D-5L sub-scale scores were significantly higher (worse) when the patient suffered from both a comorbidity and a complication (Table 3).

**Table 3:** EQ-5D-5L sub-scales scores by comorbidity and complication status.

**EQ-5D-5L sub-scale**	**Total, mean (SD)**	**No comorbidities or complications, mean (SD)**	**Only one comorbidity or complication, mean (SD)**	**Both comorbidities and complications, mean (SD)**	***p*-values compared to no comorbidity or complication**
Mobility	1.9 (1.1)	1.7 (1.0)	1.7 (1.1)	2.2 (1.2)	<0.001
Self-care	1.3 (0.7)	1.2 (0.6)	1.2 (0.5)	1.4 (0.9)	<0.001
Usual activities	1.4 (0.9)	1.3 (0.7)	1.3 (0.7)	1.7 (1.1)	<0.001
Pain/discomfort	1.8 (1.1)	1.7 (1.0)	1.6 (0.9)	2.1 (1.2)	<0.001
Anxiety/depression	1.6 (1.0)	1.5 (0.9)	1.5 (0.9)	1.8 (1.1)	<0.001
VAS	74.3 (20.4)	77.1 (18.7)	77.5 (17.1)	68.6 (23.8)	<0.001

(Sub-scales scoring: 1 = no problems, 5 = extreme problems) and (VAS score: 0 = worst health imaginable, 100 = best health imaginable).

SD, standard deviation; VAS, visual analog scale.

Table 4 presents the EQ-5D-5L sub-scales by two categories: patients who did not report a problem with the sub-scale (scored 1) and patients who reported any problem with the sub-scale (scored 2, 3, 4, 5). Similarly, patients who had both a comorbidity and complication were significantly more likely to report a problem in all sub-scales.

**Table 4:** EQ-5D-5L subscales categories by comorbidity and complication status.

**EQ-5D-5L sub-scale**	**Category**	**Total, *N* (%)**	**No comorbidities or complications, *N* (%)**	**Only one comorbidity or complication, *N* (%)**	**Both comorbidities and complications, *N* (%)**	***p*-values**
Mobility	No problem	643 (54.5%)	127 (67.6%)	342 (60.1%)	174 (41.1%)	<0.001
	Any problem	537 (45.5%)	61 (32.4%)	227 (39.9%)	249 (58.9%)	
Self-care	No problem	998 (84.6%)	166 (88.3%)	509 (89.5%)	323 (76.4%)	0.002
	Any problem	182 (15.4%)	22 (11.7%)	60 (10.5%)	100 (23.6%)	
Usual activities	No problem	890 (75.9%)	151 (81.2%)	465 (82.4%)	274 (64.9%)	<0.00
	Any problem	282 (24.1%)	35 (18.8%)	99 (17.6%)	148 (35.1%)	
Pain/discomfort	No problem	661 (56%)	111 (59%)	363 (63.8%)	187 (44.2%)	0.002
	Any problem	519 (44%)	77 (41%)	206 (36.2%)	236 (55.8%)	
Anxiety/depression	No problem	778 (65.9%)	131 (69.7%)	407 (71.5%)	240 (56.7%)	0.007
	Any problem	402 (34.1%)	57 (30.3%)	162 (28.5%)	183 (43.3%)	

Patients with neither comorbidities nor complications had higher (better) VAS scores (6.8, 95% CI: 3.3–10.3; *p* < 0.001) than patients with both comorbidities and complications, as shown in Table 5. Similarly, patients with only one comorbidity or complication had an increased (better) VAS score (6.9, 95% CI: 4.2–9.5; *p* < 0.001) compared to patients with both comorbidities and complications. On the other hand, female patients showed a decrease in VAS score (2.8, 95% CI: 5.4–0.3; *p* = 0.026), indicating a worse level of perceived health than males.

**Table 5:** VAS score regression model.

	**Parameter**	***B***	**95% confidence interval**		***p*-value**
			**Lower**	**Upper**	
Gender	Female^a^	−2.8	−5.4	−0.3	0.026
Complication and comorbidity status	No comorbidities or complications^b^	6.8	3.3	10.3	<0.001
	Only one comorbidity or complication^b^	6.9	4.2	9.5	<0.001
Insulin	Does not take insulin^c^	4.2	1.8	6.7	0.001
Education	Diploma/university^d^	7.7	3.9	11.5	<0.001
	Elementary/intermediate/secondary^d^	5.5	1.8	9.2	0.004
(Intercept)		70.5	65.3	75.6	<0.001

B, unstandardized coefficient.

a Reference category “Male.”

b Reference category “Both comorbidities and complications.”

c Reference category “Take insulin.”

d Reference category “Illiterate.”

Education level also showed a difference in VAS scores, with increased education levels associated with higher (better) VAS scores. Patients with a higher level of education scored higher (7.7, 95% CI: 3.9–11.5; *p* < 0.001) than illiterate patients, and patients with a middle level of education scored higher (5.5, 95% CI: 1.8–9.2; *p* = 0.004) than illiterate patients. Moreover, Taking insulin had a negative effect on VAS score. Patients who did not take insulin assessed their overall health as better (4.2, 95% CI: 1.8–6.7; *p* = 0.004) than those who use insulin.

Table 6 shows that being female was associated with a negative effect on all EQ-5D-5L sub-scales. Gender had the highest impact on usual activities; female patients were more likely to score worse on the usual activities sub-scale compared to male patients (OR = 3.377, 95% CI: 2.2–5.2; *p* < 0.001).

**Table 6:** EQ-5D-5L sub scales regression models.

	**Mobility**		**Self-care**		**Usual activities**		**Pain/discomfort**		**Anxiety/depression**	
	**OR**	**CI**	**OR**	**CI**	**OR**	**CI**	**OR**	**CI**	**OR**	**CI**
Female^a^	2.1^***^	(1.5–2.8)	2.1^**^	(1.3–3.4)	3.4^***^	(2.2–5.2)	1.9^***^	(1.5–2.5)	1.6^***^	(1.2–2.1)
≥66 years old^b^	2.2^***^	(1.5–3.4)	2.1^*^	(1.1–3.8)	1.9^*^	(1.1–3.0)			0.7	(0.5–1.0)
51–65 years old^b^	1.5^*^	(1.1–2.1)	1.3	(0.8–2.3)	1.1	(0.7–1.7)			0.6^**^	(0.5–0.9)
No comorbidities or complications^c^	0.6^*^	(0.4–0.9)	0.7	(0.4–1.3)	0.6^*^	(0.3–1.0)	0.6^**^	(0.4–0.8)	0.5^**^	(0.4–0.8)
Only one comorbidity or complication^c^	0.6^***^	(0.4–0.8)	0.4^***^	(0.3–0.6)	0.5^***^	(0.3–0.7)	0.5^***^	(0.4–0.6)	0.6^***^	(0.4–0.7)
Does not take insulin^d^	0.7^**^	(0.5–0.9)					0.8^*^	(0.6–1.0)	0.5^***^	(0.4–0.7)
Obese (BMI ≥30)^e^	1.8^***^	(1.4–2.4)	1.6^*^	(1.0–2.4)	1.5^*^	(1.0–2.2)				
Diploma/university^f^	0.5^**^	(0.3–0.8)	0.3^***^	(0.2–0.6)	0.5^*^	(0.3–0.9)				
Elementary/intermediate/secondary^f^	0.6^*^	(0.4–0.9)	0.4^***^	(0.2–0.6)	0.5^**^	(0.3–0.8)				
Duration of disease (years)							1.0^*^	(1.0–1.0)		

OR, odds ratio; CI, confidence interval.

* p < 0.05.

** p < 0.01.

*** p < 0.001.

a Reference category “Male.”

b Reference category “50 years old and younger.”

c Reference category “Both comorbidities and complications.”

d Reference category “Take insulin.”

e Reference category “Not obese (BMI<30).”

f Reference category “Illiterate.”

Age also had a negative impact on patients' assessment of most of the sub-scales. Patients ≥66 years of age reported more problems with mobility, self-care, and usual activities than patients <50 years old (OR = 2.2, 95% CI: 1.5–3.4, *p* < 0.001; OR = 2.1, 95% CI: 1.1–3.8, *p* < 0.05; and OR = 1.9, 95% CI: 1.1–3.0, *p* < 0.05, respectively). However, on the anxiety/depression sub-scale, there was an opposite tendency as patients aged 51–65 reported lower degree of problems with anxiety/depression than younger patients (OR = 0.6, 95% CI: 0.5–0.9, *p* < 0.01).

Patients having only one comorbidity, or one complication were more likely to score lower (better) on all sub-scales compared to those who had both comorbidities and complications. This tendency for patients with only one comorbidity or complication to score better than patients with both was especially apparent with the self-care sub-scale (OR = 0.4, 95% CI: 0.3–0.6, *p* < 0.001), the usual activities sub-scale (OR = 0.5, 95% CI: 0.3–0.7, *p* < 0.001), and the pain/discomfort sub-scale (OR = 0.5, 95% CI: 0.4–0.6, *p* < 0.001). Patients with only one comorbidity or complication were also more likely score lower (better) on the mobility and anxiety/depression sub-scales, although the impact was slightly lower (OR = 0.6, 95% CI: 0.4–0.8, *p* < 0.001, and OR = 0.6, 95% CI: 0.4–0.7, *p* < 0.001, respectively). Compared to those with both comorbidities and complications, patients without comorbidities and complications were also less likely to score higher (worse) for mobility (OR = 0.6, 95% CI: 0.4–0.9, *p* < 0.05), usual activities (OR = 0.6, 95% CI: 0.3–1.0, *p* < 0.05), pain/discomfort (OR = 0.6, 95% CI: 0.4–0.8, *p* < 0.01), and anxiety/depression (OR = 0.5, 95% CI: 0.4–0.8, *p* < 0.01) sub-scales.

Those who do not take insulin were less likely to score higher (worse) than those who do take insulin on mobility (OR = 0.7, 95% CI: 0.5–0.9, *p* < 0.01), pain/discomfort (OR = 0.8, 95% CI: 0.6–1.0, *p* < 0.05), and anxiety/depression (OR = 0.5, 95% CI: 0.4–0.70, *p* < 0.001) sub-scales. Similarly, being obese led to a statistically significant increase in the likelihood of scoring higher (worse) on mobility (OR = 1.8, 95% CI: 1.4–2.4, *p* < 0.001), self-care (OR = 1.6, 95% CI: 1.0–2.4, *p* < 0.05), and usual activities (OR = 1.5, 95% CI: 1.0–2.2, *p* < 0.05) sub-scales.

The higher a patient's level of education, the lower (better) were the scores on the sub-scales. Compared to illiterate patients, those with an elementary/intermediate/secondary education were more than twice as likely to score lower (better) on the self-care (OR = 0.4, 95% CI: 0.2–0.6, *p* < 0.001) and usual activities (OR = 0.5, 95% CI: 0.3–0.8, *p* < 0.01) sub-scales. Patients with an elementary/intermediate/secondary education were also more likely to score lower (better) on the mobility sub-scale (OR = 0.5, 95% CI: 0.4–0.9, *p* < 0.05). Having a higher education level (diploma/university) also made patients much more likely to score lower (better) on the mobility (OR = 0.5, 95% CI: 0.3–0.8, *p* < 0.01), self-care (OR = 0.3, 95% CI: 0.2–0.6, *p* < 0.001), and usual activities sub-scales (OR = 0.5, 95% CI: 0.3–0.9, *p* < 0.05).

The duration of the disease did not show a significant impact on four of the five EQ-5D-5L sub-scales. The exception was pain/discomfort; each additional year of diabetes lead to an increase in the likelihood of scoring higher (more pain) in this sub-scale (OR = 1.0, 95% CI: 1.0–1.0, *p* < 0.05).

A statistically significant (*p* < 0.001) negative relationship was shown between VAS scores and all EQ-5D subscales. For example, an increase in the depression subscale score always (*p* < 0.001) led to a decrease (ρ < 0.0) in overall VAS score, but the decrease was not high (the absolute value of ρ was < 0.3).

## 4. Discussion

HRQoL related to diabetes can be difficult to measure because of its perceived subjective nature (6). For this reason, many physicians may focus more on objectives measures, such as HbA1c levels, and on treatment for complications such as cardiovascular issues, than on patient-reported HRQoL factors (7). However, a growing body of research shows that HRQoL plays an important role in disease management for many chronic diseases, including diabetes, impacting patient adherence to treatment, health outcomes, patient productivity, and patient perspective of their own emotional, mental, and social states (8).

Previous studies of the Kuwaiti population showed that people living with diabetes in Kuwait report lower HRQoL than people without diabetes. In a pediatric study published in 2013, the quality of life of Kuwaiti children and adolescents (2–18 years old) with type 1 diabetes was compared to children and adolescents without diabetes. That study revealed an HRQoL that was lower than the control group in the pediatric population (9). A study published in 2018 showed that Kuwaiti adults living with diabetes also expressed lower and progressively declining levels of overall HRQoL than people without diabetes (10). That study included primary care patients only.

In our study of adult Kuwaiti nationals living with diabetes, we sought to identify patient characteristics and variables that affect patient-perceived HRQoL. Our analysis demonstrated that the variables that led to worse VAS and EQ-5D-5L sub scale scores (indicating lower perceived HRQoL) were being female, obesity (BMI ≥30), insulin usage, lower levels of education, and the presence of complications and/or comorbidities. However, a statistical association between two variables (for example insulin usage and HRQoL score) merely implies that knowing the value of one variable (e.g., insulin usage) provides information about the value of the other (e.g., HRQoL score). It does not necessarily imply that one variable causes the other.

These results appear to align with many aspects of previous studies in Kuwait and other countries, including studies that used different patient survey instruments (7, 9, 11–15). The 2018 study of Kuwaiti adults, which used the Diabetes Self-Management Questionnaire (DSMQ) (16) and the Short Form Health Survey (SF-12) (17) instruments, showed that female patients with diabetes rated their overall and physical health components of HRQoL lower than male patients (10). Obesity and insulin usage were also demonstrated to have a negative effect on HRQoL. That study also showed that complications and comorbidities had a negative impact, with the impact growing with the number of complications and comorbidities (10). In particular, studies in other countries using the EQ-5D, DSMQ, the SF-12, or the Audit of Diabetes-Dependent Quality of Life (ADDQoL) (18) instruments frequently show a correlation between the presence of complications and/or comorbidities with a lower perceived HRQoL (11, 14, 15, 19, 20). However, some studies in other countries found conflicting results. For example, while a study in Taiwan using the ADDQoL instrument showed a negative relationship between diabetes-related complications and insulin usage on quality of life, which aligned with our study results, it showed a greater negative impact from higher education and for being male in some sub-scales, which was contradictory to our results (14). A 2020 study in Poland, the Czech Republic, and Slovakia using the ADDQoL instrument showed that three of the factors our study identified as predictors of lower HRQoL—lower levels of education, diabetes-related complications, and insulin usage—instead had a positive effect on quality of life (21). It is unclear what may cause these types of contradictory results; possible reasons could include differences in regional dietary habits, study instruments, population criteria, completeness of data, healthcare treatment, environment, socioeconomic status, lifestyle habits, or social factors (20, 22).

Some sub-scale scores in our study aligned with another study done in Norway, which also used the EQ-5D instrument (19). In that study, 25% of the patients reported problems with usual activities; in our study it was 23.9%. Pain and discomfort was a reported problem in 45% of the Norwegian patients, which was similar to the findings in our study (43.9%). Lastly, anxiety and depression were apparent in a third of both the Norwegian patients (33%) and our Kuwaiti patients (34%). However, in two other sub-scales, our study's results were almost double the Norwegian scores. Forty-five percent of our patients reported a problem with mobility; on the other hand, only 26% of the Norwegian patients had problems with mobility. Problems with self-care were reported in 15.3% of our Kuwaiti patients but in only 6% of Norwegian patients. The last two sub-scales' results may be attributed to a more sedentary lifestyle in Kuwait and the higher likelihood of Kuwaitis having paid helpers at home to help with usual activities.

However, a study done in Saudi Arabia that used the EQ-5D instrument found different results than our study (23), with a higher percentage of Saudi Arabia patients reporting problems in all sub-scales than the Kuwaiti patients in our study. The Saudi Arabia study participants reported more problems in mobility, self-care, usual activities, pain and discomfort, and depression and anxiety, than our study's participants. The study populations were significantly different between the two studies. The Saudi Arabia patients were younger. Most of the Saudi Arabia patients had normal weight, while in our study the majority were obese. These differences in population characteristics may explain the differences in the sub-scales.

Moreover, a study done in Iran looked at the factors that affected HRQoL using the EQ-5D instrument (24). They found that duration of diabetes increased the likelihood of scoring higher (worse) on mobility and usual activities, while our study showed duration of diabetes only increased the likelihood of scoring higher (worse) in the pain and discomfort domain. In the Iranian study, being female negatively affected almost all domains except for the usual activities domain. However, in our study being female negatively affected all domains. Table 7 summarizes the factors that affected HRQol negatively and positively in the above mentioned studies.

**Table 7:** Factors affecting HRQoL; summary from our study and different studies.

**Description of studies**	**Positive factors on HRQoL**	**Negative factors on HRQoL**
Our study	Higher level of education	Female—older age—taking insulin—having both a comorbidity and complication
Norwegian study (19)	None reported	Older age—presence of complications—receiving disability pension—receiving help from others
Saudi study (23)	Higher level of education—being married—having a family history of type 2 diabetes mellitus—exercising regularly—adhering to prescribed medications	Older age—working in the medical field—uninsured status
Iranian study (24)	Higher level of education—urban resident—employed patient	Female—older age—taking insulin—history of hospitalization—presence of complication—longer duration of disease

### 4.1. Strengths and limitations

A particular strength of this study was the inclusion of data from a large number of patients from all six governorates of Kuwait and from both hospitals and primary care clinics, which makes the data generalizable to the general Kuwaiti population across all provider settings. In addition, this study did explore the effects of both comorbidites and complications on HRQol in diabetes patients, these factors were not well-explored in other studies.

One of the limitations in this study that it used convenience sampling, this may have resulted in a unrepresentative sample of diabetes patients in Kuwait. We only captured patients who sought care; sicker patients who don't go to diabetes centers may have been missed. In addition, patients who are nonadherent to their treatment plan may have been missed because they may be more prone to miss their doctor's appointment. Another limitation is that, for consistency and access, this study evaluated only patient data for Kuwaiti nationals. Because non-Kuwaitis are estimated to make up 65% of the Kuwait population (25), the responses from non-citizen patients may vary due to treatment differences resulting from prescription policies for non-citizens. In addition, our study included only patients from the outpatient setting. Because the EQ-5D-5L and VAS instruments assess patients' perception of their health status on a particular day, this means our study population may score their health differently than hospitalized patients.

Quality of life is difficult to measure and has a high degree of subjectivity. The instrument we used (the EQ-5D-5L) is widely used and user-friendly, but it still has a high degree of subjectivity, as do other instruments. The results of the EQ-5D-5L from this study would be difficult to compare directly to studies that measure QoL specifically for diabetes, such as the ADDQoL.

## 5. Conclusions

For Kuwaiti nationals living with diabetes and the healthcare providers who treat them, HRQoL is a concern that merits ongoing attention. The factors that contribute to lower perceived HRQoL in Kuwait are being female, obesity (BMI ≥30), insulin usage, lower levels of education, and the presence of complications and/or comorbidities. Understanding how these factors affect a patient's perceived HRQoL can help healthcare providers and patients work together to identify ways to mitigate them. For example, treatment plans may be modified so that diabetes-related complications are controlled or minimized, the importance of treatment adherence may be emphasized, or options for addressing mental health concerns may be discussed. Better diabetes management leads to better control of diabetes and its complications and comorbidities, which can improve overall quality of life.

## Data availability statement

The raw data supporting the conclusions of this article will be made available by the authors, without undue reservation.

## Ethics statement

The studies involving human participants were reviewed and approved by Kuwait University (KU) Health Sciences Center (HSC) Ethical Committee and Ministry of Health (MOH) Ethical Committee. This study has been granted ethical approval from KU-HSC Ethical Committee on October 8th 2018, reference number (VDR/EC/3411). Also, it has been granted approval from MOH Ethical Committee on November 4th 2018, reference number (903/2018). The patients/participants provided their written informed consent to participate in this study.

## Author contributions

Conceptualization, methodology, and validation: MA and SA. Formal analysis, funding acquisition, investigation, project administration, supervision, and writing—original draft: MA. Resources: AAlad, TA, AAlat, and DA. Visualization: MA, SA, AAlad, TA, and AAlat. Writing—review and editing: MA, SA, AAlad, TA, AAlat, and DA. All authors contributed to the article and approved the submitted version.
